# Cross‐Sectional Studies: Strengths, Limitations, and Methodological Considerations

**DOI:** 10.1111/jre.70063

**Published:** 2026-01-05

**Authors:** Sebastian‐Edgar Baumeister, Thomas Kocher, Panos N. Papapanou, Birte Holtfreter, Ryan T. Demmer

**Affiliations:** ^1^ Institute of Health Services Research in Dentistry University of Münster Münster Germany; ^2^ Department of Restorative Dentistry, Periodontology, and Endodontology University Medicine Greifswald Greifswald Germany; ^3^ Division of Periodontics, Section of Oral, Diagnostic and Rehabilitation Sciences, College of Dental Medicine Columbia University New York New York USA; ^4^ Division of Epidemiology, Department of Quantitative Health Sciences Mayo Clinic College of Medicine and Science Rochester Minnesota USA

**Keywords:** cross‐sectional study, epidemiology, observational study, study design

## Abstract

Cross‐sectional studies capture health states, exposures, and risk factors at a single time point, providing essential data for estimating disease prevalence and informing public health planning. These studies serve multiple epidemiological purposes: characterizing population health, monitoring temporal trends through repeated surveys, and evaluating interventions via interrupted time series designs. They also offer practical advantages for validating self‐reported measures and creating diagnostic models. Cross‐sectional designs are efficient and well‐suited to descriptive epidemiology, but they have limited utility for causal inference. The simultaneous measurement of exposures and outcomes creates temporal ambiguity that fundamentally constrains etiologic interpretation. However, causal inferences can be strengthened under specific conditions—when temporal sequence is unambiguous (e.g., genetic variants preceding outcomes) or when valid instrumental variables are available. This methodological tutorial equips readers with concepts and tools to critically appraise cross‐sectional studies across the application domains outlined and to design and analyze their own cross‐sectional studies that yield high‐quality epidemiologic descriptions.

## Graphical abstract

Cross‐sectional studies measure variables at a single time point to estimate prevalence and support descriptive epidemiology ‐ characterizing population health, tracking trends via repeated surveys – and for developing and validating screening instruments and diagnostic models. Their simultaneous measurement of exposures and outcomes creates temporal ambiguity that fundamentally limits causal inference, though exceptions exist when temporal sequence is clear or valid instrumental variables are available.

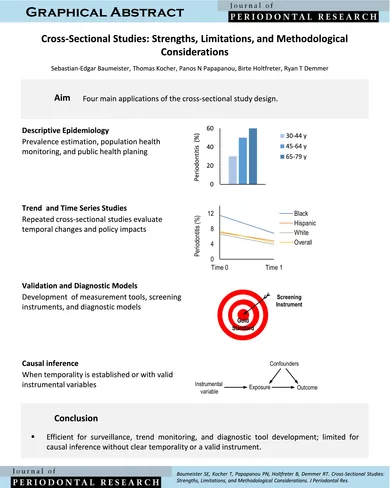

## 1 Uses of the Cross‐Sectional Design Design

The cross‐sectional design captures a population snapshot at a single time point. It assesses measurements in a one‐time sample from a dynamic population. The study base comprises person‐moments, as in cohort studies, but outcomes are ascertained without follow‐up—measuring disease presence/absence at those person‐moments [1]. This article provides coverage of the cross‐sectional study design, commonly used to describe the current state of diseases or risk factors in populations and examine temporal trends. Beyond descriptive epidemiology, this design validates measurement instruments and develops diagnostic models for disease detection and screening. The article concludes by examining the use of cross‐sectional designs for causal inference. Cross‐sectional designs are commonly applied in four domains: descriptive surveillance of diseases and risk factors; trend and time‐series analyses using repeated cross‐section studies to characterize population changes and evaluate interventions; validation studies and diagnostic model development/validation; and causal analyses when temporality is established or valid instrumental variables exist.

The following sections review each domain and highlight design‐specific strengths and limitations to guide interpretation and use. Table 1 presents a glossary of key terms and concepts (italicized). Table 2 summarizes challenges and limitations by application domain, and Table 3 presents examples of cross‐sectional studies across domains. Guidance and a checklist for reporting cross‐sectional studies are provided by the STROBE (Strengthening the Reporting of Observational Studies in Epidemiology) guideline [17].

**TABLE 1:** Glossary of key terms and concepts used in cross‐sectional studies.

Analytical sample	The subset of study participants included in the analysis after applying eligibility criteria and handling missing data
Autocorrelation (serial correlation)	The correlation of a variable with itself across time; observations close in time tend to be more similar than distant ones
Backdoor path	A non‐causal path from exposure to outcome in a directed acyclic graph that remains after removing all arrows pointing from the exposure to other variables
Bias (systematic error)	A consistent deviation of an estimate from the true causal effect due to flaws in design, data, or analysis (e.g., confounding or selection bias)
Calibration	Agreement between predicted probabilities and observed outcome rates; assessed with calibration plots, calibration intercept/slope, observed/expected ratio, or the Brier score
Census	Data collection that attempts to include every member of the entire population (no sampling)
Clinical utility	The extent to which using a diagnostic model improves decision‐making or patient care in practice
Collider	A variable that is a common effect of two other variables
Collider bias	Bias introduced by conditioning on a collider (e.g., selecting, adjusting, or stratifying), which opens a noncausal path between exposure and outcome
Complex sampling	A random sampling design that uses stratification, clustering, and/or unequal selection probabilities (often multistage). Requires survey weights and design‐based variance estimation
Confounder	A variable that opens a backdoor path in a directed acyclic graph
Confounding	Distortion of the exposure–outcome relationship caused by one or more confounders creating an open backdoor path
Convenience sample	A nonprobability sample selected based on ease of access (e.g., volunteers, clinic attendees), which may not represent the target population
Diagnostic model	A model that combines predictors (e.g., symptoms, signs, tests, risk factors) to estimate the current probability of a condition
Directed acyclic graph (DAG)	A diagram with one‐way arrows representing assumed causal relationships among variables, used to identify confounders, mediators, and colliders and to guide adjustment
Discrimination	The ability of a diagnostic model to distinguish people with the outcome from those without it; commonly summarized by the area under the ROC curve (AUC)
Exchangeability (instrumental variable assumption)	No open backdoor path between instrument and outcome
Exclusion restriction (instrumental variable assumption)	The instrument affects the outcome only through its effect on the exposure, with no direct or alternative pathways
External validation	Testing a diagnostic model in new data not used to develop it (e.g., from a different time, place, or population) to assess performance
Generalizability	The extent to which study findings or a model's performance apply to other populations, settings, or times beyond the one studied
Gold standard reference	The best available method for determining true disease status, against which other tests or models are compared
Goodness‐of‐fit	The agreement between observed data and values predicted by a model; for example, a pseudo‐R^2^ from a logistic regression model
Imputation	Replacing missing values with plausible values based on other observed information (e.g., single or multiple imputation) to reduce bias and loss of precision
Instrumental variable	A variable that influences the outcome only through the exposure
Interrupted time series design	A design with many observations before and after a clearly timed intervention. The intervention's effect is estimated as a change in level and/or slope after the interruption
Inverse probability weighting	A method for adjustment for selection bias, for nonrandomly missing data, or for differences among exposure groups in the estimation of causal effects (adjustment for measured confounders)
Matching	A design and analysis method that pairs or groups participants with similar values of confounders across exposure groups to reduce confounding
Measurement error	The difference between a true value and the measured value of variable
Mediation analysis	Methods to decompose the total effect of an exposure on an outcome into direct and indirect effects through a mediator, requiring correct temporal ordering and control of confounding
Mediator	A variable on the causal pathway from exposure to outcome; adjusting for it blocks part of the causal effect
Multivariable regression	A statistical technique that simultaneously adjusts for multiple confounding variables to estimate the association between an exposure and outcome, or uses multiple predictor variables to model and predict an outcome
Post‐stratification	A common weight adjustment in complex samples where weights are modified so that weighted totals within strata (e.g., age, sex) match known population totals
Prevalence	The proportion of a population with a condition at a specific point in time (point prevalence) or over a defined period (period prevalence)
Random sample	A probability sample in which each member of the target population has a known, nonzero probability of selection; in a simple random sample, all members have an equal selection probability
Randomized trial	An experimental study in which participants are randomly assigned to exposure or treatment, creating exchangeable groups and minimizing confounding
Recalibration	Adjusting a diagnostic model (e.g., intercept and/or slope or coefficients) to improve calibration in a new dataset or setting
Repeated cross‐sectional design	Independent random samples from the same target population are drawn at multiple time points to track population‐level trends; different people are measured at each time
Representative sample	A random sample that resembles a target population of interest
Selection bias	(i) Systematic differences between those included in the study or analysis and the target population due to the selection process (e.g., clinic‐based sampling, nonresponse, volunteer bias), leading to distorted prevalence estimates. (ii) Bias arising when inclusion in, or analysis of, the study depends on variables related to exposure and outcome (often via conditioning on a collider), leading to distorted estimates
Standardization	A weighting‐based method to remove the effect of differences in variables like age when comparing two or more populations (e.g., direct or indirect standardization)
Stratifying (stratification)	Dividing the sample into subgroups (strata) based on a variable (e.g., age group, sex) for analysis
Survival bias (survivor bias)	Bias that arises because only individuals who are alive or remain in the sample at the survey time are observed, which can underrepresent severe cases or harmful exposures
Target population	The full group about which the study aims to make inferences (the population to whom findings are intended to apply)
Time series	A sequence of observations on the same unit or aggregate measured at regular intervals (e.g., annually). Enables analysis of patterns such as long‐term trends and seasonality
Trend study	Research focused on changes over calendar time often using repeated cross‐sectional or time‐series data
Validation	The process of evaluating a model's performance (e.g., calibration and discrimination) in data from the same study or in independent data
Weighting (survey weights)	Assigning weights to participants to account for selection probabilities, nonresponse, and to align the sample with known population totals; used in estimation and variance calculations

**TABLE 2:** Challenges and limitations of the cross‐sectional study design by application domain.

	Type of bias or error	Challenges	Limitations
Descriptive study	Selection bias	Representativeness and selection: Convenience samples and nonresponse can produce selection bias	Analytical sample (after exclusions/missing data) may not reflect the target population
	Statistical precision	Complex sampling design: stratification, clustering, and weights	Assuming a simple random sample can bias estimates and standard errors
	Selection bias	Prevalence as the estimand: Prevalence reflects incidence, duration, and survival	Length‐biased sampling and survival bias can distort prevalence estimates, especially for chronic diseases
	Selection bias	Comparability across groups: Standardization can aid comparisons but may obscure real differences attributable to adjusted variables	Unequal demographic distributions across cross‐sectional samples can obscure or distort between‐group differences in diseases or risk factors
Trend and time‐series studies	Selection bias, measurement error	Consistency in sampling frames, disease definitions, and measurement approaches across time points	Shifts in sampling frames, case definitions, or measurement methods across cross‐sectional samples can generate spurious trends
	Selection bias	Varying age or other covariate distributions across waves can bias trend estimates if not standardized	Unequal age and sex distributions across cross‐sectional samples can distort real trends
	Invalid counterfactual trend, confounding	Pre‐intervention trajectory would have continued unchanged absent the intervention. No other policy changes, shocks, seasonality shifts	Concurrent interventions or shocks. Unmodeled seasonality or calendar effects. Staggered or gradual implementation. Spillovers and substitution effects
Validation and diagnostic model studies	Measurement error	Imperfect “gold standard”	Biased and inaccurate performance metrics. Misleading decision analysis
	Generalizability	Diagnostic models predict current disease status only	The performance of a cross‐sectional diagnostic model cannot be extrapolated to its predictive or prognostic performance
	Generalizability	External validation, generalizability assessment, and potential recalibration are required before implementation	Development‐sample performance often overestimates real‐world performance
	Generalizability	Good discrimination/calibration alone do not ensure clinic impact	Effectiveness and utility require further evaluation (e.g., impact study or trials)
Causal inference in cross‐sectional studies	Temporality	Exposure, outcome, and confounders measured at the same time hinder establishing temporality	Variables may be confounders, mediators, or colliders, but cannot be reliably distinguished
	Selection bias	Prevalent‐case design overrepresent long‐duration/surviving cases	Length‐biased sampling and survival bias distorts exposure–outcome associations
	Temporality	Mediator must be measured after exposure and before outcome, which is rarely attainable in purely cross‐sectional designs	With a cross‐sectional design, decomposing effects into direct and indirect components is often not feasible
	Confounding	Reliable confounding adjustment requires longitudinal data with temporally ordered measurements of confounders, exposure, and outcomes	With cross‐sectional data, pre‐exposure confounders cannot be reliably distinguished from post‐exposure variables, increasing the chance of adjusting for mediators or colliders
	Selection bias, temporality, confounding	Approaches like an instrumental variable design require stringent assumptions (exclusion restriction, exchangeability); cross‐sectional cohort design depends on accurately reconstructed histories	A valid instrumental variable may be unavailable, and assumptions may fail. Differential participation/selection in the cross‐sectional sample can undermine causal inference even when confounders, exposures, and outcomes are reconstructed and temporally aligned

**TABLE 3:** Examples of the cross‐sectional study design by application domain.

Application domain	Characteristics^a^	Examples
Descriptive study	*Population*: adult civilian population; *Sampling*: complex *Condition*: periodontitis, diabetes	United States: National estimates of common dental and medical conditions using the National Health and Nutrition Examination Survey (NHANES) [2, 3].
	Population: adult civilian population; Sampling: complex Condition: periodontitis, caries	Germany (West Pomerania): Regional prevalence estimation using the Study of Health in Pomerania (SHIP) [4].
	*Population*: adult civilian population; *Sampling*: complex *Condition*: hypertension	England: Hypertension and diabetes prevalence in adults using the Health Survey for England [5].
Trend and time‐series studies	*Population*: school population; *Sampling*: complex *Condition*: multiple oral diseases	United Kingdom: Tracking caries trends among children using successive Children's Dental Health Survey (CDHS) waves [6].
	*Population*: adult civilian population; *Sampling*: complex *Condition*: caries, edentulism	Brazil: Using SB Brazil national oral health surveys to track trends in caries and edentulism across survey waves [7].
	*Population*: adult civilian population; *Sampling*: complex *Outcome*: blood lead level	United States: Decline in blood lead levels after the phase‐out of leaded gasoline using NHANES repeated cross‐sections and interrupted time‐series/trend analyses [8].
Validation and diagnostic model studies	*Population*: adult civilian population; *Sampling*: complex *Gold standard*: periodontitis *Screening tool*: questionnaire	United States: Development of a periodontitis screening instrument using full‐mouth periodontal examination (clinical attachment loss/pocket depth) as a gold standard [9].
	*Population*: adult civilian population; *Sampling*: cluster *Outcome*: oral cancer *Predictors*: age, sex, tobacco/alcohol use, and lesion features	South Asia: Community oral cancer screening study in Sri Lanka using multivariable logistic regression [10].
	*Population*: adult civilian population; *Sampling*: complex *Outcome*: type 2 diabetes, abnormal glucose tolerance, metabolic syndrome *Predictors*: age, body mass index, waist circumference, diet, antihypertension medication, blood glucose, family history	Finland: FINDRISC diabetes risk score; cross‐sectional development and validation of a multivariable questionnaire‐based model to detect undiagnosed type 2 diabetes and impaired glucose regulation in primary care and population surveys [11].
Causal inference in cross‐sectional studies	*Population*: adult civilian population; *Sampling*: complex *Outcome*: depression, cardiovascular disease, labor market participation *Exposure*: caries, tooth loss *Instrumental variable*: water fluoridation	United States: Community water fluoridation status/coverage or fluoride concentration as instrument variables for caries/tooth retention to estimate effects on depression, cardiovascular disease, or labor market outcomes [12, 13, 14].
	*Population*: adult civilian population; *Sampling*: complex *Outcome*: tooth loss, dental visits *Exposure*: schooling years *Instrumental variable*: schooling reforms	United States/Europe: Using schooling reforms as instrumental variables for education to estimate causal effects on tooth loss and dental visits [15].
	*Population*: U.S. veterans; *Sampling*: convenience (health records) *Outcome*: tooth loss, dental visits *Exposure*: low‐ versus high opioid prescription *Instrumental variable*: random provider assignment	United States: Using arbitrary assignment of patients to providers as instrumental variable to assess effect of low‐intensity versus high‐intensity opioid prescriptions [16].

^a^ e.g. population, sampling design, condition, outcome, treatment/exposure, instrumental variable.

## 2 Descriptive Studies

The primary application of the cross‐sectional design is describing population health status to inform public health surveillance and planning. A descriptive study begins with a clearly articulated, well‐defined question specifying: (1) the target population, characterized by person and place, anchored in time; (2) the outcome, event, or health state; and (3) the occurrence measure used to summarize the outcome (e.g., *prevalence*) [18, 19]. For instance, a study might estimate the periodontitis prevalence among adults in the United States (U.S.) on a specific date (December 1, 2014) or over a defined period (the calendar year 2014). Answering such questions typically involves a population survey—a descriptive cross‐sectional study utilizing a *random sample* drawn from the target population. The target population, defined as the source population from which the study random sample is drawn, comprises all individuals for whom the outcome distribution is intended to be estimated (Figure 1A). The definition of this target population is intrinsically linked to the study's objective, such as estimating disease burden to plan for healthcare needs or preventive services. Unlike a random sample, the target population is not necessarily enumerated, but its membership must be defined in terms of person, place, and time [1, 20]. In the periodontitis example, this might include all U.S. residents aged 18 years or older on December 1, 2014, or during January 1–December 31, 2014.

**FIGURE 1:**
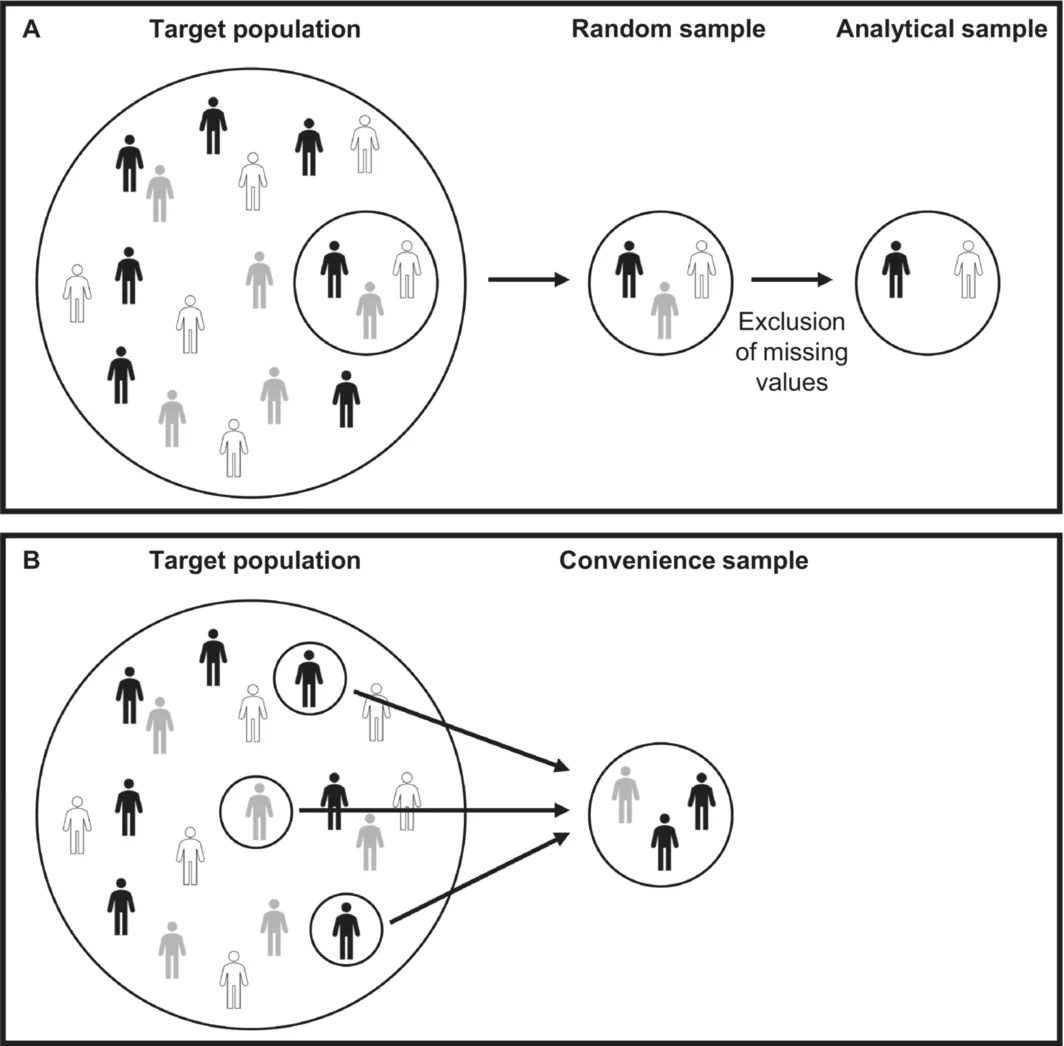
Schematic representation of random and convenience samples from target population. The target population is the entire population about which the cross‐sectional study seeks to make inferences. A random (probability) sample enables unbiased estimation of population parameters such as prevalence. However, the analytical sample—after excluding records with missing data—may yield biased estimates.

When full *census* data are available (e.g., from administrative records or public health surveillance), random sampling is unnecessary. Otherwise, we rely on *representative samples* from the target population. A random sample is a subset where each individual has a known, non‐zero selection probability [20]. In practice, many descriptive studies rely on *convenience samples* (e.g., patients at a specific hospital) and implicitly assume that this sample is representative of the target population (Figure 1B). However, convenience samples can differ systematically from the target population—particularly when participation is related to the outcome of interest or its determinants. In such cases, *selection bias* can arise. This form of bias distorts descriptive estimates when inclusion in the analytical sample is associated with the outcome [21]. The *analytical sample* refers to the subset of participants actually used in the analysis, typically after excluding individuals with missing data (Figure 1A). When using this analytical sample to estimate *prevalence*, researchers generally assume that it is a random sample of the target population, with an identical distribution of the outcome [20, 21]. If this assumption is violated, but the sample can be considered conditionally random given certain measured covariates, statistical adjustments are possible. Methods such as *post‐stratification*, *weighting*, *standardization*, and *imputation* can help correct the resulting bias. These techniques often leverage external data from the target population (e.g., census data) to produce estimates that more accurately reflect the true parameter in the target population [20, 21].

The estimand in descriptive analyses—such as the prevalence of a disease—is defined as a function of outcomes that occur in the entire target population. This design enables the estimation of prevalence, which refers to the proportion of existing cases at a specific point in time (point prevalence) or over a defined period (period prevalence), rather than newly occurring incident cases. Disease prevalence ascertained at one point in time reflects a combination of disease incidence, duration, and survival [22, 23]. For example, a rare but long‐lasting condition may have a relatively high prevalence, while a common but short‐lived or highly fatal disease may show low prevalence. In addition, *survival bias* can affect estimates when considering prevalent outcomes, particularly in chronic diseases. Thus, even descriptive analyses must consider the temporal structure underlying the observed data, which arise from a dynamic population [1, 24, 25]. Nevertheless, while prevalence is not an optimal measure of disease occurrence, it remains an appropriate—though suboptimal—measure for chronic, incurable conditions with long duration and uncertain onset [26]. In cross‐sectional studies, prevalence is often the only available information to support public health decision‐making and care planning.

When elaborating on prevalence, it is often informative to stratify individuals with the outcome by key characteristics such as age, sex, gender, or race/ethnicity. *Stratifying* on multiple characteristics can be informative, especially when guided by conceptual models highlighting important stratifying factors. Alternatively, researchers may apply statistical adjustments to account for nuisance variables that cause the observed prevalence to deviate from that of the target population or from other populations used for comparison. A common example is age standardization, which adjusts a sample's age distribution to match that of a reference or target population. This adjustment facilitates more equitable comparisons across populations and can help identify health disparities. However, while standardization is a useful tool, it may sometimes obscure real differences between populations—such as differences between ethnic or geographic groups—when those differences are genuinely attributable to the adjusted variables [27].

Returning to estimating U.S. adult periodontitis prevalence, the National Health and Nutrition Examination Survey (NHANES)—a repeated cross‐sectional survey monitoring population health since 1960—provides key data [2]. NHANES employs *complex sampling* with subpopulation oversampling for nationally representative estimates. Analysis requires specialized statistical procedures accounting for stratification, clustering, and weighting, with post‐stratification ensuring alignment with U.S. census population totals [20]. Using CDC and American Academy of Periodontology surveillance definitions on 2009–2014 cycles, Eke and colleagues estimated 42.2% of U.S. dentate adults aged 30–79 had periodontitis (7.8% severe, 34.4% moderate/mild) [2]. Prevalence was significantly higher among males, older adults, current smokers, lower socioeconomic groups, Hispanics, and non‐Hispanic blacks. State‐level estimates showed substantial geographic disparities (37.7% in Utah versus 52.8% in Mexico), with the greatest burden in southeastern and southwestern states, the Mississippi Delta, and U.S.–Mexico border regions. Such cross‐sectional surveillance studies provide essential public health policy and clinical practice data. These estimates enable monitoring progress toward objectives like Healthy People 2020's goal to reduce moderate/severe periodontitis from 47.2% (2009–2010 NHANES) to 40.8% (2020) in the U.S. [28]. Identifying high‐risk populations facilitates targeted prevention, while geographic variation guides resource allocation and region‐specific program design. Establishing baseline prevalence using standardized definitions creates foundations for evaluating long‐term trends.

## 3 Trend and Time‐Series Studies

Descriptive studies portraying current health status can be extended to examine population disease and risk factor trends over time. Major cross‐sectional surveys—such as NHANES—are conducted at regular or irregular intervals, allowing researchers to estimate aggregate or population‐level temporal changes. These surveys provide current population estimates at each time point and detect changes or trends since previous rounds. This approach, known as a *trend study*, consists of serial cross‐sectional studies conducted over time to evaluate changes in population health status. An ‘independently pooled cross‐section’ is created by drawing new, random samples from the same target population at different time points. For example, the British Association for the Study of Community Dentistry Survey annually examines new samples of children and adolescents to monitor dental caries prevalence.

Long‐term trend data inform public health policy and guide targeted prevention strategies. Based on analyses of repeated cross‐sectional studies in high‐income countries, overall periodontal health has improved only modestly over the last three decades [4]. Specifically, severe periodontitis prevalence has remained largely unchanged across most studies. However, this period has seen minor increases in moderate periodontitis prevalence in the U.S. Borrell et al. [29] showed that severe periodontitis prevalence among U.S. adults decreased from 7.3% in NHANES III (1988–1994) to 4.2% in NHANES 1999–2000. This decline occurred across all racial/ethnic groups, with non‐Hispanic blacks showing the largest absolute reduction (from 11.4% to 6.8%), followed by Mexican‐Americans (6.9% to 4.6%) and non‐Hispanic whites (6.7% to 3.8%).

In standard *repeated cross‐sectional design*, independent cross‐sectional random samples are selected each time, meaning minimal or no overlap exists among individuals surveyed across waves. This contrasts with longitudinal or cohort studies, which analyze individual‐level change by following the same participants through successive follow‐ups. A core assumption is that each survey wave comprises independently sampled observations, minimizing statistical correlation between individuals across time periods. When incidental or intentional sample overlap occurs—as in rotating panel designs—*autocorrelation* depends on the overlap extent and individual characteristic stability over time. A key concern is maintaining consistent sampling methods to ensure each wave's representativeness of the target population at its specific time point, yielding unbiased estimates. When repeated cross‐sections include wider age ranges and age distributions (and other characteristics) vary across cross‐sections, covariate distributions can be aligned via standardization.

Beyond descriptive monitoring, repeated cross‐sectional data provide stronger causal evidence, particularly when evaluating policy impacts or large‐scale events. For example, NHANES data documented declining population blood lead levels following regulatory lead removal from gasoline [8]. This analysis type, using pooled cross‐sections to evaluate population‐level interventions, is formalized as *interrupted time series* (ITS) analysis [30]. In ITS design, pre‐intervention repeated cross‐sections establish the underlying pre‐intervention trend. This trend is statistically projected forward to estimate the counterfactual—what would have occurred without the intervention. The observed post‐intervention trends are then compared to projected counterfactual trends. Significant deviations between observed and projected trends indicate intervention effects. For example, ITS analysis assessed the effect of reduced public dental coverage on dental care utilization in the Netherlands [31]. Reporting of trend studies should follow the GATHER (Guidelines for Accurate and Transparent Health Estimates Reporting), the National Center for Health Statistics Guidelines for Analysis of Trends, and STROBE guidelines [17, 32, 33]. For ITS and other quasi‐experimental studies using repeated cross‐sectional data, reporting should draw on the TREND statement, Cochrane EPOC guidance, and related resources [34, 35, 36].

## 4 Validation and Diagnostic Model Studies

Cross‐sectional designs are also well suited for validating measurement instruments. When a study simultaneously collects data using both a potentially error‐prone tool and a corresponding *gold standard reference*, the degree of *measurement error* can be estimated and quantified. For example, researchers may conduct a study to assess the validity of self‐reported questionnaire items used for periodontitis surveillance or screening [9]. Moreover, these studies can facilitate the development of *diagnostic models* that integrate self‐reported data with socioeconomic, behavioral, and clinical risk factors. The goal of such models is to improve the discrimination between individuals at low and high risk for a condition, such as periodontal disease. A practical application of this is the Diabetes Risk Assessment in Dentistry Score, which was specifically created to help identify patients at high risk for undiagnosed diabetes in dental care settings [37].

The creation of such diagnostic models typically follows a rigorous, multi‐step process. This process generally includes: (1) conducting a systematic review and meta‐analysis to identify established disease predictors; (2) selecting candidate predictors based on these findings; (3) employing appropriate statistical methods for final variable selection; and (4) comprehensively evaluating the final model's performance, including assessments of *goodness‐of‐fit*, discrimination, calibration, and *clinical utility* [38]. Traditional approaches select predictors via univariable screening and stepwise procedures guided by *p*‐values or information criteria (AIC/BIC), but these strategies are unstable and prone to overfitting. More recent practice emphasizes penalized methods (ridge, lasso, elastic net) and machine‐learning models with embedded selection, tuned via resampling or nested cross‐validation to optimize out‐of‐sample performance [39]. Stability selection and Bayesian approaches address model uncertainty. Regardless of statistical method, pre‐specifying predictors based on subject‐matter knowledge is recommended [38, 39]. Crucially, it must be emphasized that the development of a diagnostic model is only the initial step. Before any such tool can be recommended for routine clinical or public health screening, it must undergo extensive further evaluation. This critical subsequent phase includes rigorous *external validation*, assessment of *generalizability* to different populations, potential model updating or *recalibration* for new settings, and ultimately, testing of effectiveness and efficacy through randomized trials [40]. Diagnostic accuracy studies should be reported according to STARD (Standards for Reporting Diagnostic Accuracy) and its extensions [41, 42]. Prediction model studies should follow TRIPOD (Transparent Reporting of a multivariable prediction model for Individual Prognosis or Diagnosis) and its extensions [43, 44, 45].

## 5 Causal Inference in Cross‐Sectional Studies

Randomized trials are the gold standard for causal research, but many causal questions must be answered with observational data. In a well‐designed observational study, causal inference uses design and analysis tools to estimate the effect of an exposure on an outcome, guided by a prespecified causal structure. Causal diagrams (*directed acyclic graphs*, or DAGs) help map prior knowledge, identify variables to adjust for, and flag variables that should not be adjusted [21, 46]. In a DAG, arrows represent assumed causal effects, paths connect variables, the target path runs from exposure to outcome, and *backdoor paths* represent noncausal routes that can bias the association.

*Bias* (systematic error) distorts exposure–outcome associations. *Confounding* arises when a common cause affects both the exposure and the outcome, opening a backdoor path. For example, when studying whether physical activity reduces periodontitis, smoking may influence both and confound the association (Figure 2A). Confounding can often be removed by adjusting for a “minimal” set of variables that closes all backdoor paths; sometimes adjusting for a proxy (e.g., education in place of unmeasured socioeconomic status) can help if it sufficiently blocks the path (Figure 2B). A *mediator* lies on the causal pathway from exposure to outcome (Figure 2C). Because adjusting for a mediator blocks part of the causal effect, mediators should not be adjusted for when estimating the total effect. A *collider* is a common effect of two variables. Paths through a collider are closed by default, but conditioning on a collider (e.g., by restricting to a selected sample) opens a spurious path and induces collider (or selection) bias. For instance, early studies in the coronavirus pandemic suggested that smoking might protect against Covid‐19 infection [47]. It turned out that these observations were likely due to collider bias because study participants were selected from hospitals and hospitalization was influenced by both smoking‐related disease and COVID‐19, distorting the observed association (Figure 2D). Bias can be mitigated at the design stage and in analysis. Common confounding‐adjustment methods include *multivariable regression*, *matching*, and *inverse probability weighting*; *inverse probability weighting* and *imputation* strategies can also help address selection bias.

**FIGURE 2:**
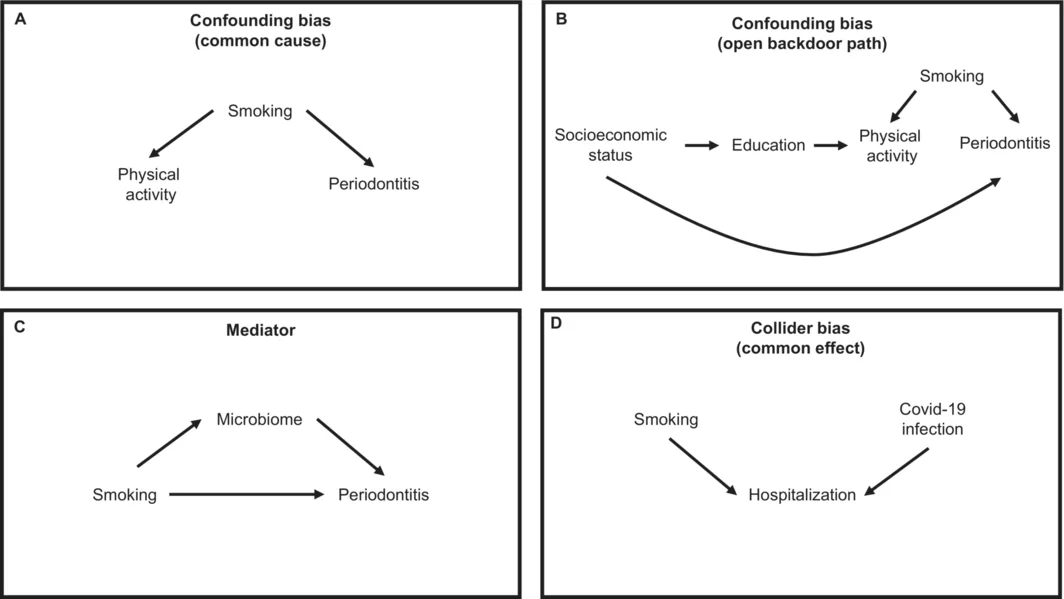
Directed Acyclic Graphs illustrating confounding, mediation and collider bias. The figure uses directed acyclic graphs (DAGs) to illustrate: (A) confounding by a common cause of the exposure and outcome, inducing a spurious association; (B) confounding via an unblocked backdoor path from exposure to outcome; (C) decomposition of the total effect into direct and indirect (mediated) components; and (D) collider bias introduced by conditioning on a variable influenced by both exposure and outcome (e.g., restricting the sample to one level of that variable).

Cross‐sectional studies pose special challenges for causal inference because exposure and outcome are measured at the same time. Causes must precede effects, and confounders must be measured before the exposure to plausibly influence it (and the outcome) [48, 49]. When variables are measured simultaneously, it is often unclear whether they are confounders, mediators, or colliders. As a result, longitudinal data with temporally ordered measurements of confounders, exposures, mediators, and outcomes are generally needed for reliable confounding adjustment and for mediation analysis [25, 26, 50, 51]. *Mediation analysis* requires mediators to be measured after the exposure and before the outcome; this timing is rarely attainable in purely cross‐sectional designs, making them unsuitable for mediation analysis [52, 53, 54, 55].

There are exceptions. Some exposures are fixed and clearly precede outcomes (e.g., germline genetic variants or parental education). Cross‐sectional studies can also collect retrospective histories of exposures and outcomes to reconstruct timing (“cross‐sectional cohort” designs) [22, 56, 57]. Another strategy is the *instrumental variable* (IV) approach. An IV affects the exposure, influences the outcome only through that exposure (*exclusion restriction*), and is not confounded with the outcome (*exchangeability*) [21, 46, 58] (Figure 3A). For instance, cigarette prices and smoke‐free laws can serve as IVs for smoking when estimating its effect on tooth loss in cross‐sectional data, assuming these policies affect tooth loss only by altering smoking behavior and are otherwise independent of factors that cause tooth loss (Figure 3B) [59]. Swanson and Hernán [60] proposed a reporting checklist for IV studies.

**FIGURE 3:**
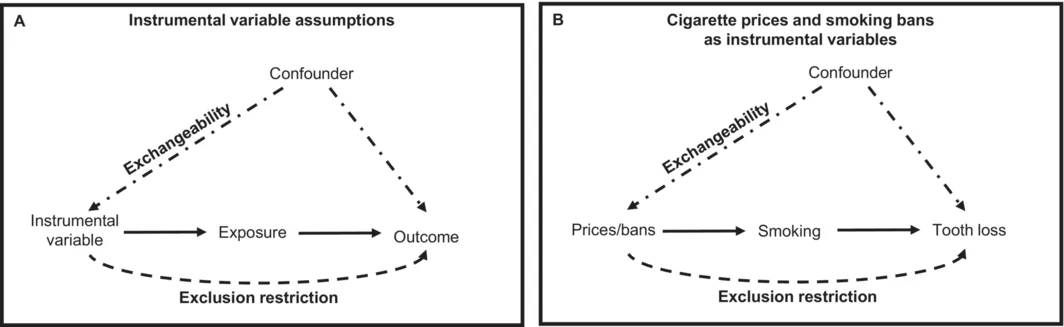
Instrumental variable assumptions and application to study the effect of smoking on tooth loss. The figure illustrates the instrumental variable assumptions for estimating the causal effect of smoking on tooth loss. A valid instrument (i.e., cigarette price/bans) must affect tooth loss only through smoking (exclusion restriction) and have no unblocked backdoor path to the outcome (no instrument–outcome confounding).

## 6 Conclusion

The primary advantage of cross‐sectional studies is that they offer one of the most practical feasible and efficient means for studying nonfatal diseases. Cross‐sectional studies can be incorporated into ongoing surveillance programs, enabling the screening of impaired health, monitoring of adverse exposures, and description of population health status. When conducted repeatedly, they allow for the characterization of population‐level disease trends, which are valuable for public health monitoring, planning, and evaluating the impact of interventions or exogenous events affecting entire populations. Additionally, cross‐sectional designs can support the development of screening and diagnostic tools. By contrast, cross‐sectional data are generally ill‐suited to causal inference because exposure and outcomes are measured at a single time point, undermining temporality requirements.

Cross‐sectional studies will continue serving essential functions in epidemiology and public health. Their role in surveillance, burden estimation, and trend monitoring remains irreplaceable for non‐fatal conditions and population health assessment. However, researchers should recognize these designs as primarily descriptive tools, reserving causal questions for longitudinal studies with appropriate temporal ordering of measurements. When cross‐sectional data are used for causal inference, explicit acknowledgment of assumptions and limitations is essential.

## Funding

The authors have nothing to report.

## Conflicts of Interest

The authors declare no conflicts of interest.

## Supporting information


**Data S1:** jre70063‐sup‐0001‐supinfo.docx.

## Data Availability

The data that support the findings of this study are available on request from the corresponding author. The data are not publicly available due to privacy or ethical restrictions.
