# Supplementary material for: Cross‐Sectional Studies: Strengths, Limitations, and Methodological Considerations
Source: J Periodontal Res. 2026 Jan 5;61(7):749–60. doi: 10.1111/jre.70063 (PMC13665586; doi:10.1111/jre.70063)
Supplement: Supplementary file 1 [file JRE-61-749-s001.docx]

**Social Media Document**

- Please indicate below the Instagram account name for each study author.
- These accounts will be tagged if the study is shared on the *Journal of Periodontal Research* official Instagram account ***(@J.Perio.Research***) to enhance visibility and reach.

|  | **Author Name/Surname** | **Instagram Account(s) Name** |
| --- | --- | --- |
| **1** | Sebastian-Edgar Baumeister | No Instragram Account |
| **2** | Thomas Kocher | No Instragram Account |
| **3** | Panos N Papapanou | **@panos.papapanou** |
| **4** | Birte Holtfreter | No Instragram Account |
| **5** | Ryan T Demme | No Instragram Account |
| **6** |  |  |
| **7** |  |  |
| **8** |  |  |

- If an author does not have an Instagram account or does not want to be tagged, please enter “No account” in the corresponding cell.

____________________________________________________________________

**Social Media Statement**

*This statement will serve as the post's caption. It should be concise, engaging, rigorous and designed to generate interest in the study. It will be peer-reviewed.*

Cross-sectional studies are a cornerstone of descriptive epidemiology and disease surveillance, helping track the occurrence and trends of diseases and risk factors in populations.
